# Trypanocidal Effects of Cisplatin alone and in Combination with *Nigella sativa* Oil on Experimentally Infected Mice with *Trypanosoma evansi*

**Published:** 2018

**Authors:** Nashaat Abd El-Monem NASSEF, Manal Ahmed EL-MELEGY, Engy Victor BESHAY, Dalia Rifaat Al-SHARAKY, Tahany Mohamed AL-ATTAR

**Affiliations:** 1. Medical Parasitology Department, Faculty of Medicine, Menoufia University, Menoufia, Egypt; 2. Pathology Department, Faculty of Medicine, Menoufia University, Menoufia, Egypt

**Keywords:** *T. evansi*, Mice, Cisplatin, Diminazene, *N. sativa*, Trypanocidal

## Abstract

**Background:**

Due to the limited number of the available drugs for the treatment of trypanosomiasis, this study was designed to evaluate the trypanocidal effects of cisplatin or/and *Nigella sativa* oil (NSO) in experimentally infected mice with *T. evansi*.

**Methods:**

During 2015 at the Parasitology Department, Menoufia University, Menoufia, Egypt, sixty Swiss albino mice were divided into six groups: normal control (I), infected control (II); cisplatin-treated (III); NSO-treated (IV); combined cisplatin + NSO-treated (V) and diminazene-treated (VI). The tested drugs were evaluated by the assessment of parasitaemia, measurement of aspartate aminotransferase (AST), alanine aminotransferase (ALT), urea, creatinine, serum IgM and a histopathological study.

**Results:**

NSO showed a trypanocidal effect, however; it was not as effective as cisplatin or diminazene. There were significant increases of AST, ALT, urea, and creatinine in group II and III, which were significantly reduced in cisplatin + NSO-treated group (V). Moreover, there were significant reductions in serum IgM and the pathological changes of the examined organs of group V when they were compared with other treated groups.

**Conclusion:**

Cisplatin combined with NSO showed a trypanocidal effect against *T. evansi* with preservation of vital organs functions and architecture.

## Introduction

Trypanosomes are parasitic protozoans that live in the blood of a great variety of vertebrate hosts and are transmitted by an arthropod intermediate host (1). One of these trypanosomes is *T. evansi*, which can affect either humans or animals throughout the tropical and subtropical areas (2).

The current chemotherapy of human African trypanosomiasis relies on only six drugs (Suramin, pentamidine, melarsoprol, eflornithine, arsobal and Mel B). While other drugs such as homidium, isometamidium, and diminazene aceturate are used for the treatment of infected animals. Each of these drugs has one or more of challenges: being highly toxic, its need for parenteral administration and the risk of emergence of drug resistance (3).

Drug ‘repurposing’ is the identification of new therapeutic applications for drugs received US FDA approval for another purpose (4). One of these drugs is cisplatin [cis-diaminedichloro-platinum (II)] (5) which is an antitumor DNA binding drug that was found to have anti-leishmanial activities. However, the toxicity reported with cisplatin treatment motivated some studies for the prevention of this toxicity via the simultaneous supplementation of herbal extracts (6). Among the medicinal herbs *Nigella sativa* is emerging as a miracle herb with anti-inflammatory (7), cytoprotective (8), immunomodulatory (9) and antiparasitic (10) effects.

Therefore, this study aimed to evaluate the trypanocidal effect of cisplatin alone and combined with *N. sativa* oil (NSO) in experimentally infected mice with *T. evansi* by assessment of parasitaemia, biochemical assays, serological and histopathological studies.

## Material and Methods

### Experimental animals

This study was approved by the Scientific Research Ethical Committee, Faculty of Medicine Menoufia University, Menoufia, Egypt where the study was conducted during June 2015 at the Parasitology Department. Swiss female albino mice aged 60 d and weighing on average 25 ± 0.2 g were kept under controlled temperature and humidity (25 °C; 70%) and fed with commercial ration and water *ad libitum*.

### Isolation, identification of T.evansi and preparation of the infective inoculum

Fresh blood infected with *T. evansi* was obtained from naturally infected camels. Wet blood smears were made to detect the motile trypanosomes. The parasite was identified by its morphological features. In fresh unstained blood smears, it showed thin posterior end, free actively moving anterior flagellum and a highly visible undulating membrane (11). Giemsa-stained thin smears were made (12), and the dominating monomorphic slender forms of average 24±4 μm length were identified as *T. evansi* by its characteristic long free flagellum, an undulating membrane, thin posterior extremity and the subterminal small kinetoplast. Infected blood was immediately inoculated intraperitoneally into two mice, each with 0.2 ml. Inoculated mice were examined by wet blood smears and once parasitaemia had established, the donor mice were anesthetized, 1 ml of blood was collected, diluted with glucose phosphate buffered saline and the experimental inoculum was adjusted to contain 10^6^ trypanosomes/0.2 ml (13).

### Experimental design

The experimental mice were divided into six groups (10 mice each): The control group (I) received 0.2 ml physiological saline intraperitoneally, the infected non-treated group (II) received 0.2 ml of infected blood intraperitoneally, Cisplatin (Cisplatine)**,** (MYLAN S.A.S., France) treated group (III) received a dose of 3mg/kg/d intraperitoneally for five successive days starting on day 2 p.i.(14), NSO (El-Nile Co. for pharmaceuticals and chemical industries) treated group (IV) received a dose of 5 mg/kg/d orally for five successive days starting three days before infection (10), Combined cisplatin + NSO-treated group (V) and finally, Diminazene aceturate (BATRYNIL, Arab Company for Medical Products) treated group (VI) received a dose of 7 mg/kg once intraperitoneally on day 2 p.i. (11).

Fifteen days post-infection, all mice were sacrificed. From each mouse, blood was collected; serum was obtained by centrifugation at 3000 rpm for 10 min then stored at −80 °C until required for biochemical and serological studies. Moreover, the liver, spleen, and brain were removed, fixed in 10% formalin for a histopathological study.

### Assessment of parasitaemia

Thin blood films were made from each mouse, fixed, stained and examined under a microscope. Parasitaemia was monitored every other day until the 14^th^ post-infection day by counting the number of parasites present in ten microscopic fields at 1000X magnification (15).

### Biochemical assays

Commercially available kits (Sigma-Aldrich^®^, USA) were used to measure serum ALT and AST activities and the values were expressed as international unit/liter (IU/L). Additionally, blood urea and creatinine were measured and the values were expressed as mg/dl (16).

### Enzyme immunoassay test (EIA)

For estimation of serum IgM levels (ng/ml), all serum samples were tested using ENZO (USA) IgM (mouse), EIA kit Catalog No. ADI-900-120.

### Histopathological study

The formalin-fixed organs from each mouse were embedded in paraffin then serial transverse sections (5 μm in thickness) were prepared and stained with hematoxylin and eosin (Hx&E) (17).

### Statistical analysis

The collected data were tabulated and analyzed by SPSS (ver. 20, Chicago, USA). ANOVA (f-test) and Kruskal-Wallis test (k-test) were used then followed by a post-hoc test to determine significance between groups. *P*<0.05 was considered significant.

## Results

### Parasitaemia

**Fig. 1:**
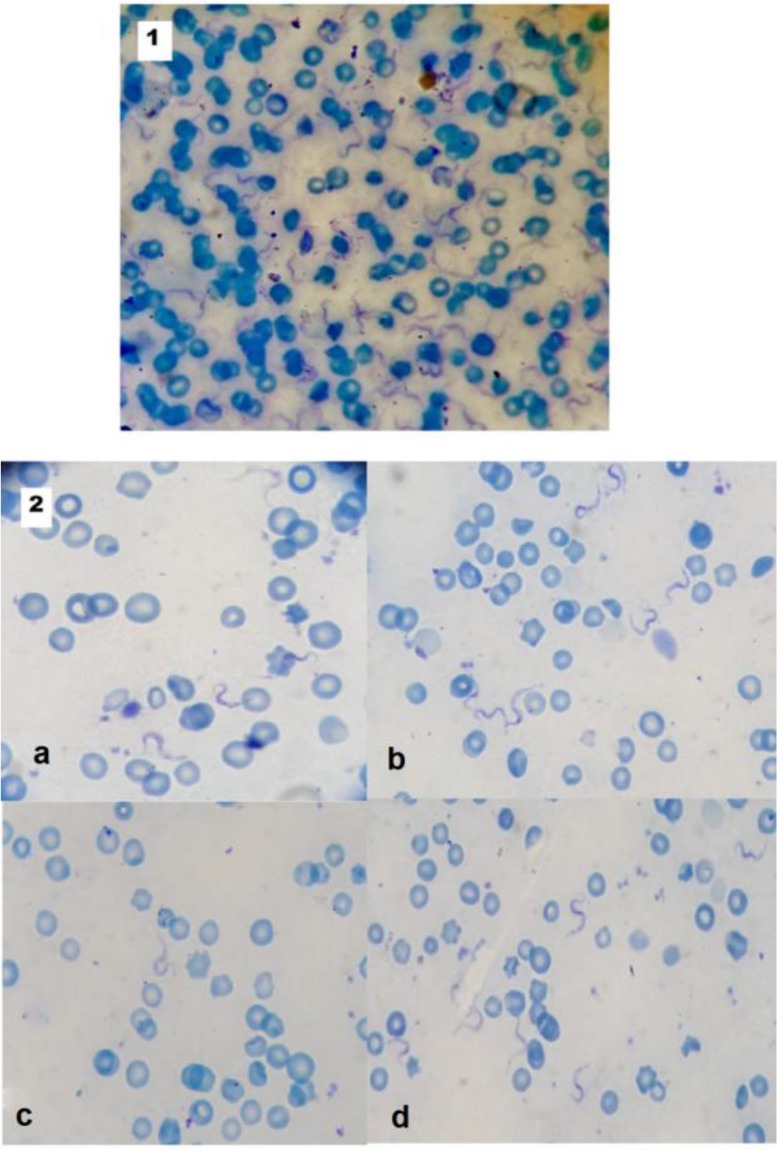
Giemsa-stained thin blood film from *T. evansi* infected control group (II) showing many trypomastigotes *(1)*. Thin blood films from infected and treated groups showing reduction of parasitaemia in cisplatin-treated (a), NSO-treated (b), combined cisplatin + NSO-treated (c) and diminazene-treated (d) (2) (Giemsa, 1000X)

**Fig. 2:**
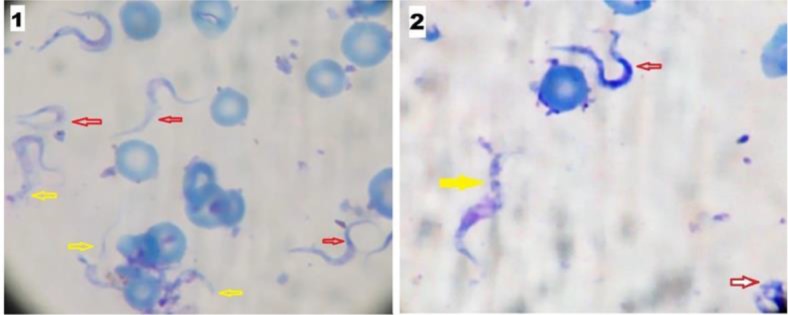
Thin blood film from the cisplatin-treated group at 6^th^ p.i.d. showing trypomastigotes which had lost its undulant membranes (red arrows) and ruptured trypomastigotes (yellow arrows) *(1).* Thin blood film from combined cisplatin + NSO treated group at 4^th^ p.i.d. showing trypomastigotes which had lost its undulant membranes (red arrow), ruptured trypomastigotes (yellow arrows) and bizarre form (white arrow) *(2)* (Giemsa, 1000X, zoom was used)

The peak of parasitaemia was recorded on the 4^th^ post-infection day in the infected control (group II) then it declined gradually. All the treated groups showed significant reductions in parasitaemia (*P*<0.001) when they were compared with group II. Both cisplatin and NSO showed trypanocidal effects, however, NSO alone was not as effective as cisplatin or diminazene thus the difference was significant between cisplatin-treated group (III), cisplatin + NSO treated group (V) and diminazene treated group (VI) in comparison with NSO-treated group IV (Table 1, Fig.1). Moreover, Giemsa-stained thin blood films from group III, V and VI revealed morphological changes of *T. evansi* trypomastigotes (Fig. 2).

**Table 1:** The parasitaemia values among the different studied groups

***Groups Parasitaemia***	***Group II***	***Group III***	***Group IV***	***Group V***	***Group VI***	***Test***	***P-value***
2^nd^ P.i.d.	32.3±5.4^d^	30.2±0.63^d^	30.6±1.7	27.8±1.4^abce^	31.2±0.92^d^	**f=**4.22	< 0.05
4^th^ p.i.d.	54.0±8.7	15.6±1.2^ac^	20.3±4.2^a^	11.6±1.3^abc^	7.8±0.81^abc^	**f=**175.8	<0.001
6^th^ P.i.d.	48.8±18.5	1.9±0.58^ace^	8.9±2.9^a^	1.4±0.51^ace^	2.5±0.23^ac^	**k=**37.4	<0.001
8^th^ P.i.d.	18.1±6.8	1.35±0.26^ac^	5.5±0.25^a^	1.46±0.2^ac^	1.51±0.26^ac^	**f=**26.2	<0.001
10^th^ P.i.d.	17.7±5.6	0.9±0.09^ac^	4.7±0.25^a^	0.82±0.09^ac^	0.85±0.07^ac^	**f=** 29.1	<0.001
12^th^ P.i.d.	15.6±1.65	0.61±0.15^ac^	4.2±1.03^a^	0.54±0.16^ac^	0.7±0.82^ac^	**k=** 38.4	<0.001
14^th^ P.i.d.	12.2±7.4	0.32±0.1^ace^	3.4±1.1^a^	0.0±0.0^aca^	0.56±0.56^ac^	**k=** 35.8	<0.001

Data were expressed as mean ± SD, n = 10, f = ANOVA test, K = Kruskal Wallis. All superscripts indicate significance at *P*<0.05 (

a compared to G2,

b compared to G3,

c compared to G4,

d compared to G5,

e compared to G6)

### Serum levels of AST and ALT

The serum levels of AST and ALT were significantly higher in the infected group (II) than in the normal control group (I). The values were reduced to nearly normal in NSO-treated group (IV) and diminazene treated group (VI), thus, there was no significant difference (*P*>0.05) between those groups and group I. The highest values were recorded in the cisplatin-treated group (III) and their improvements were detected in the cisplatin + NSO-treated group (V). In the comparison of group III and group V with all other groups, the differences were significant (*P*<0.001) (Table 2).

**Table 2:** Serum AST, ALT, urea, creatinine and IgM values of the studied groups

	***Group I***	***Group II***	***Group III***	***Group IV***	***Group V***	***Group VI***	***Test***	***P-value***
AST	6.2±2.3	12.5±4.2^ᶰ^	90.1±9.6^ᶰacde^	8.8±2.6	53.0±5.35^ᶰabce^	8.6±3.1	**k=47.4**	<0.001
ALT	18.2±4.8	23.7±4.3^ᶰ^	46.4±9.9^ᶰacde^	21.8±2.1	35.1±3.0 ^ᶰabce^	21.3±2.7	**f=43.6**	<0.001
Urea	18.3±3.2	27.7±4.1^ᶰ^	84.8±21.5^ᶰacde^	28.5±2.9^ᶰ^	56.3±4.7^ᶰabce^	29.5±2.9^ᶰ^	**f=70.6**	<0.001
Creatinine	0.7±0.2	0.9±0.1^ᶰ^	2.3±0.2^ᶰacde^	0.9±0.1^ᶰ^	1.7±0.2^ᶰabce^	1.1±0.1^ᶰ^	**f=167.1**	<0.001
IgM	22.0±4.2	260.5±20.2^ᶰ^	150.0±21.4 ^ᶰac^	188.5±10.6 ^ᶰ^^a^	140.0±21.2^ᶰac^	153.5±23.6^ᶰac^	**f=180.5**	<0.001

Data of AST, ALT (U/L), urea, creatinine (mg/dl) and IgM (ng/ml) were expressed as mean ± SD, n = 10, f = ANOVA test, K = Kruskal Wallis. All superscripts indicate significance at *P*<0.05 (

ᶰ compared to G1,

a compared to G2,

b compared to G3,

c compared to G4,

d compared to G5,

e compared to G6)

### Serum levels of urea and creatinine

In all the infected groups, urea and creatinine levels showed significant differences (*P*<0.001) when they were compared with the normal control group (I). The highest values were recorded in the cisplatin-treated group (III) and their improvements were detected in the cisplatin + NSO-treated group (V). In the comparison of group III and group V with all other groups, the differences were significant (*P*<0.001) (Table 2).

### Immunoglobulin M serum level

Serum IgM levels were significantly reduced (*P*<0.001) in all the treated groups when they were compared with the infected control group (II). However, these reductions were significant; they were still significantly higher than the normal values of group I. Interestingly, among the treated groups; the lowest IgM value was of the cisplatin + NSO-treated group (V) (Table 2).

### Histopathological results Liver

**Fig. 3:**
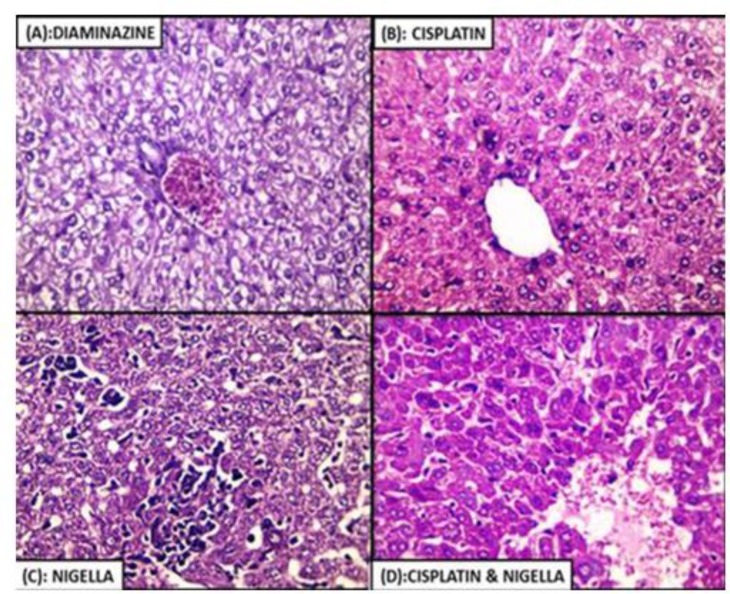
Histopathological examination of liver tissue from (A) Diminazene-treated, (B) cisplatin-treated, (C) NSO-treated, (D) cisplatin + NSO-treated groups (Hx & E, 400X)

The liver of cisplatin-treated group (III) showed minimal lymphocytic infiltrate and minimal degenerative changes. In NSO-treated group (IV), the liver showed severe lymphocytic infiltrate, loss of the normal architecture, extensive vacuolar degeneration of hepatocytes and dilated sinusoids. In the cisplatin + NSO-treated group (V), it showed minimal lymphocytic infiltrate, an absence of the degenerative changes, normally calibrated sinusoids and restoration of the normal architecture. While in diminazene-treated group (VI), it showed minimal lymphocytic infiltrate but the extensive degenerative changes were still present (Fig. 3).

### Spleen

**Fig. 4:**
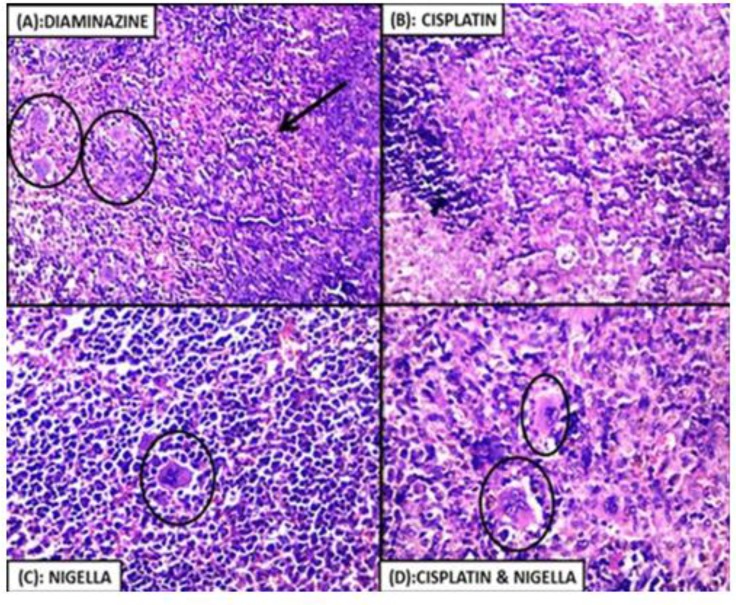
Histopathological examination of spleen tissue showing Multinucleated giant cells (black circles) with congested blood sinusoids (black arrow) in diminazene-treated group (A), NSO-treated group (C) and cisplatin + NSO-treated group (D), while cisplatin-treated group (B) showed depletion of the multinucleated giant cells (Hx & E, 400X)

Congested red pulps in all the treated groups were evident in variable degrees. The spleen of the NSO-treated group (IV) and diminazene-treated group (VI) showed extensive congestion while in the cisplatin-treated group (III) and the cisplatin + NSO-treated group (V), the spleen revealed minimal congestion. Multinucleate giant cells were observed together with congested blood sinusoids in group IV, V and VI while in group III there was a depletion of these cells (Fig. 4).

### Brain

**Fig. 5:**
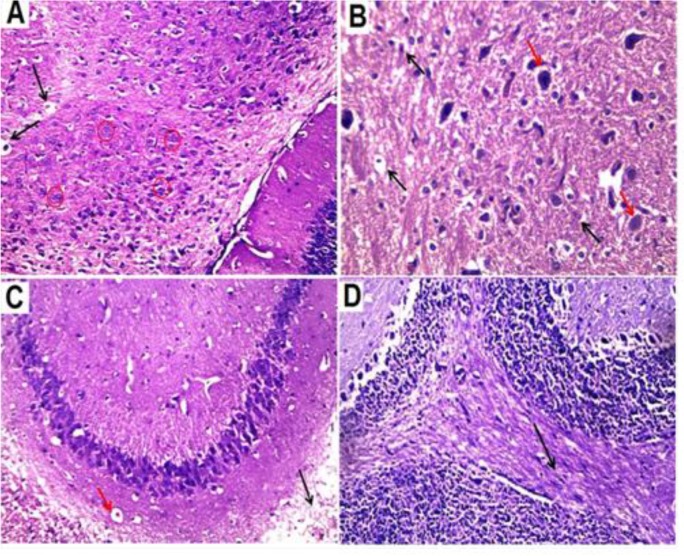
Histopathological examination of brain tissue from (A) Cisplatin-treated group showing eosinophil cell necrosis (black arrows) and relatively normal neuroglial cells (red circles) (200X), (B) NSO-treated group showing pyknotic nuclei (black arrows), vacuolation of the neuropil and basophil cell necrosis with punctate deposits at the surface (red arrows) (400X), (C) Diminazene-treated group showing intramyelinic edema (black arrow) and eosinophilic cell necrosis (red arrow), (200X) and (D) cisplatin + NSO-treated group showing evident diminished intramyelinic edema (black arrow) (Hx & E, 100X)

The brain tissues from all the treated groups showed pathological changes. In cisplatin-treated group (III) (Fig. 5A), NSO-treated group (IV) (Fig.5B) and diminazene-treated group (VI) (Fig. 5C), it revealed intramyelinic oedema, basophil neuronal necrosis, neuropil vacuolation and pyknotic basophilic nucleus. While in cisplatin + NSO-treated group (V), the brain tissue exhibited a noticeable decrease in the intramyelinic edema (Fig. 5D).

## Discussion

Due to the limited number of the available drugs for the treatment of trypanosomiasis, this study was designed to evaluate the trypanocidal effect of cisplatin alone or combined with NSO in experimentally infected mice with *T. evansi* in a comparison with the currently used drug, diminazene. The results of this study revealed a trypanocidal effect of cisplatin that was proved by the significant reduction of parasitaemia and the recorded morphological changes in Giemsa-stained blood films which were consistent with previous studies (14, 18).

We found also a trypanocidal effect of NSO, however; it was not as effective as cisplatin or diminazene. Although *N. sativa* is considered as an immunostimulating agent that potentiates both cellular and humoral immune responses (9) and an antiparasitic (10) agent, it could not eradicate the trypanosomes when it was used alone. Similar findings were reported in a study on using NSO for the treatment of *Plasmodium berghei* (10) and in a study on experimental toxoplasmosis (19). However, in our study, cisplatin + NSO treatment has resulted in the strongest trypanocidal effect.

In this study, the highest ALT and AST values were recorded in the cisplatin-treated group denoting the occurrence of hepatotoxicity, this result was in accordance with a previous study in which cisplatin was used to treat murine visceral leishmaniasis (20). Although cisplatin is significantly taken up by the liver, hepatotoxicity is a rare side effect of it. Thus, the increase in hepatic enzymes which was more pronounced in the cisplatin-treated groups could be attributed to the structural and functional derangement of the liver caused by the parasite itself then it was aggravated by cisplatin (20).

An improvement of liver enzymes in the cisplatin + NSO-treated group was found in this study declaring a hepatoprotective effect of NSO as recorded previously (8). This could be attributed to thymoquinone (an active ingredient of *N. sativa*) that was able to delay the onset and prevent the progression of the cisplatin-induced hepatotoxicity (21).

In the current study, serum urea and creatinine values of all the infected groups showed highly significant differences when they were compared with the normal control group and the highest values were recorded in the cisplatin-treated group. The results obtained with NSO were in accordance with another study (22), in which a nephroprotective effect of NSO and its ability to reduce the elevated values when it was used with cisplatin were found. In contrary to our results, in a previous study (23), the increased serum urea and creatinine levels associated with cisplatin-induced nephrotoxicity did not decrease after treatment with *N. sativa* although there was a histopathological relative recovery.

In the current study, serum IgM levels were significantly reduced in all the treated groups when they were compared with the infected control group; however, these values were still higher than the normal. Among the treated groups, the lowest IgM value was recorded in the cisplatin + NSO-treated group in association with the lowest parasitaemia level while the opposite was recorded in the NSO-treated group. These results were in harmony with previous studies which demonstrated that humoral immune system is an essential element for the protection against trypanosomiasis (24). Also, parasitaemia during the early stage of infection is mainly controlled by high IgM levels (25).

Concerning the histopathological results, there are two mechanisms which could explain those caused by *T. evansi* in the infected control group. One is the tissue-specific change and the other is the overall immunological reactions (26). *T*. *evansi* is known to utilize glucose and oxygen for its growth and multiplication, which finally leads to degenerative changes in the host’s organs. Further changes in these organs are also caused by the toxins released by the parasite (27). In the present study, the pathological findings detected in the infected control group were in accordance with previous studies (28, 29).

Regarding the pathological changes in the liver, the results of this work were consistent with a previous study (30) in which cisplatin treatment resulted in degenerative changes in hepatocytes with wide areas of necrosis and inflammatory cellular infiltrates, however, an improvement occurred after treatment of rats with methanolic neem leaves extract. Furthermore, the hepatoprotective effect of NSO on cisplatin-induced liver damage in the present work was in agreement with a previous study (20).

The spleen of all the infected groups showed depletion of the white pulps, congestion of red pulps and multinucleated giant cells infiltrates which are indicative of an immunological response caused by *T. evansi* infection (28) in variable degrees according to the response to treatment. For instance, *N. sativa* and diminazene-treated groups showed extensive congestion in contrary to the cisplatin and cisplatin + NSO-treated groups.

In this study, the improvement of the histopathological changes and restoration of the normal architecture of liver and spleen tissues that was noticed in the cisplatin + NSO-treated group could be attributed to the antioxidant properties of *N. sativa* (7, 10). These results were in agreement with some studies conducted on the administration of cisplatin alone or combined with some herbal extracts for the treatment of *L. donovani* infection in mice (5, 6). In the diminazene-treated group, the liver showed extensive degenerative changes and the spleen showed extensive congestion with multinucleate giant cells infiltrates despite diminazene was an effective trypanocidal drug. This could be explained as diminazene shows toxic effects even at the therapeutic doses (31), also it is extensively distributed in the body of the treated animals (32), and its residues may persist for several weeks, particularly in the liver and kidneys but to a lesser extent in the brain (33).

Regarding the pathological changes in the brain tissues, the results of the infected control group were similar to those reported previously (29). Interestingly, all the treated groups except the cisplatin +NSO-treated group showed pathological changes in brain tissues. The diminazene-treated group showed pathological changes similar to those recorded in the infected control group despite the strong control of parasitaemia. This could be explained by the inability of diminazene to penetrate brain tissue (32) so the parasites persisted in the brain. Although cisplatin was a potent trypanocidal agent, there were numerous pathological changes in the brain which could be attributed to its ability to cross the blood-brain barrier and induce cytotoxicity (34). While those observed with NSO treatment could be explained by its inability to eradicate the infection. However, the administration of cisplatin + NSO resulted in a marked improvement of the recorded pathological findings associated with the eradication of parasitaemia.

## Conclusion

Using cisplatin at a low dose and for a short duration combined with NSO to treat experimental *T. evansi* infection resulted in the control of parasitaemia and amelioration of the infection-induced pathological changes in the vital organs with preservation of their functions and architecture. Therefore, it is possible to take the advantage of this cytotoxic drug as a trypanocidal agent and get rid of its cytotoxicity by its combination with NSO. However, further studies should be conducted to assess the effects of administration of different doses on different immunological responses in order to clarify in more details how these drugs could control infection and modulate the host immune response.
